# Author Correction: Distinct circadian mechanisms govern cardiac rhythms and susceptibility to arrhythmia

**DOI:** 10.1038/s41467-021-27498-9

**Published:** 2021-12-08

**Authors:** Edward A. Hayter, Sophie M. T. Wehrens, Hans P. A. Van Dongen, Alessandra Stangherlin, Shobhan Gaddameedhi, Elena Crooks, Nichola J. Barron, Luigi A. Venetucci, John S. O’Neill, Timothy M. Brown, Debra J. Skene, Andrew W. Trafford, David A. Bechtold

**Affiliations:** 1 Centre for Biological Timing, Faculty of Biology, Medicine and Health, University of Manchester, Manchester, UK; 2 Faculty of Health and Medical Sciences, University of Surrey, Guildford, UK; 3 Sleep and Performance Research Center, Washington State University, Spokane, WA USA; 4 Elson S. Floyd College of Medicine, Washington State University, Spokane, WA USA; 5 MRC Laboratory of Molecular Biology, Cambridge, UK; 6 Department of Biological Sciences, Center for Human Health and the Environment, North Carolina State University, Raleigh, NC USA; 7 Unit of Clinical Physiology, Manchester Academic Health Science Centre, Faculty of Biology, Medicine and Health, University of Manchester, Manchester, UK; 8 Present Address: Department of Physical Therapy, Eastern Washington University, Spokane, WA USA

**Keywords:** Circadian regulation, Sleep deprivation, Arrhythmias

Correction to: *Nature Communications* 10.1038/s41467-021-22788-8, published online 30 April 2021.

In this article the author name John S. O’Neill was incorrectly written as John S. O’ Neill. This has now been corrected in the HTML and PDF version of this article.

