# Supplementary material for: Distinct circadian mechanisms govern cardiac rhythms and susceptibility to arrhythmia
Source: Nat Commun. 2021 Apr 30;12:2472. doi: 10.1038/s41467-021-22788-8 (PMC8087694; doi:10.1038/s41467-021-22788-8)
Supplement: Supplementary file 1 — Supplementary Information [file 41467_2021_22788_MOESM1_ESM.pdf]

Supplementary Information for

Distinct circadian mechanisms govern cardiac rhythms and susceptibility to arrhythmia

Edward A. Hayter[1], Sophie M.T. Wehrens[2], Hans P. A. Van Dongen[3,4], Alessandra Stangherlin[5], Shobhan Gaddameedhi[6], Elena Crooks[3,7], Nichola J. Barron[1], Luigi A. Venetucci[8], John S. O'Neill[5], Timothy M. Brown[1], Debra J. Skene[2], Andrew W. Trafford[1,8], David A. Bechtold[1*]

Affiliations:

[1] Centre for Biological Timing, Faculty of Biology, Medicine and Health, University of Manchester

[2] Faculty of Health and Medical Sciences, University of Surrey, Guildford, UK

[3] Sleep and Performance Research Center, Washington State University, Spokane, WA, USA

[4] Elson S. Floyd College of Medicine, Washington State University, Spokane, WA, USA

[5] MRC Laboratory of Molecular Biology, Cambridge, UK

[6] Department of Biological Sciences, Center for Human Health and the Environment, North Carolina State University, Raleigh, NC, USA

[7] Current affiliation: Department of Physical Therapy, Eastern Washington University, Spokane, WA, USA

[8] Unit of Clinical Physiology, Manchester Academic Health Science Centre, Faculty of Biology, Medicine and Health, University of Manchester

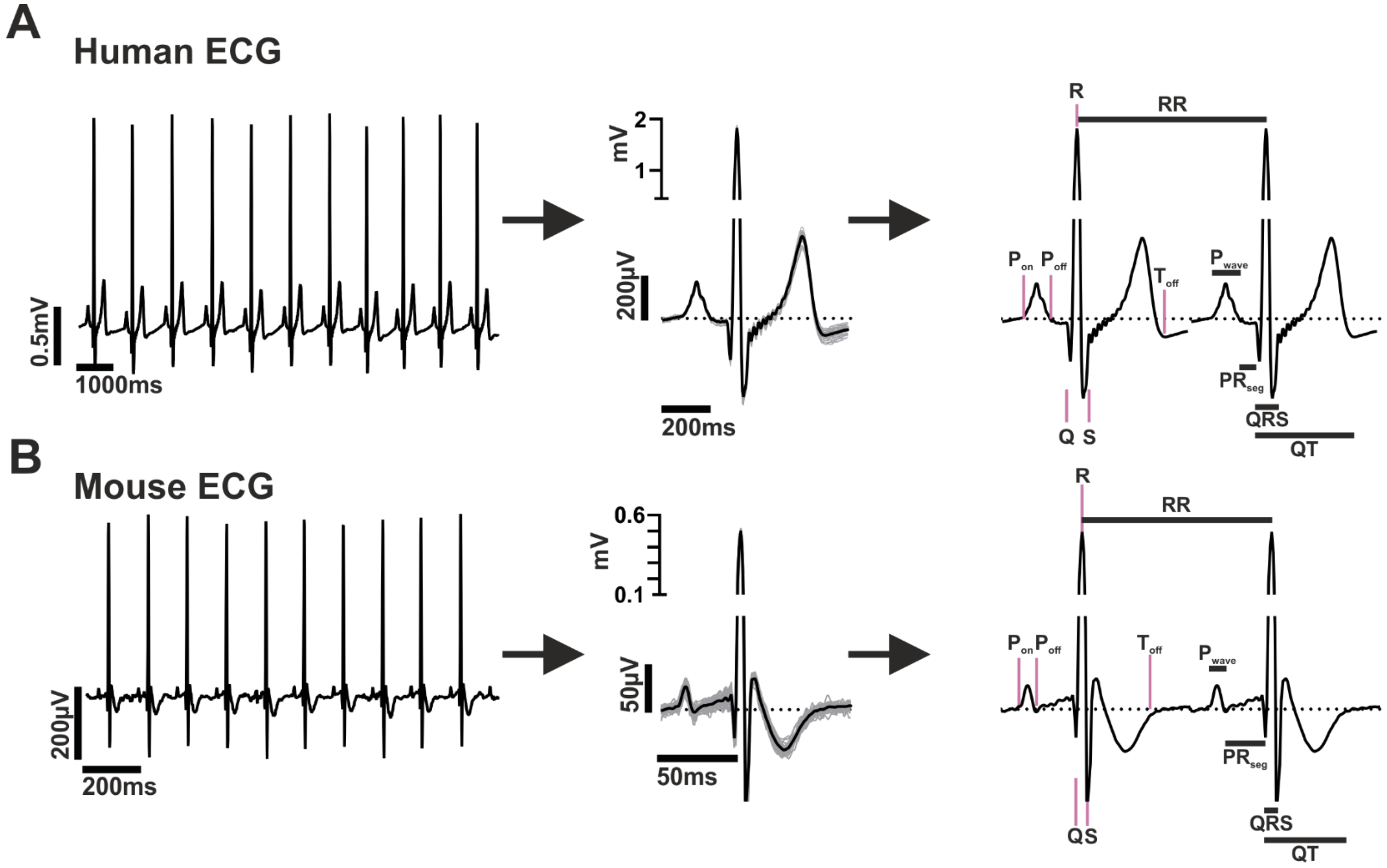

Supplementary Figure 1. Human and mouse ECG waveforms derived from longitudinal ECG recordings. In human studies, ECGs were recorded continuously via wearable devices (see methods for details). ECG features (P wave onset, offset, Q, R, S and T offset) were identified and extracted for each beat, from which mean ECG parameter values were calculated across 5-min time bins for all subsequent analyses. In mouse studies, ECGs were recorded via implanted telemetry device, with 10 sec ECG sweeps recorded every 5 min from which mean ECG parameter values were calculated. Source data are provided as a Source Data File.

RR = inter-beat interval

P_{wave} = atrial depolarisation

PR_{seg} = delay between atrial and ventricular depolarisations (conduction delay at the AV node), measured as P offset to Q onset

QRS = ventricular depolarisation

QT = ventricular depolarisation/repolarisation

HRV = Heart rate variability. HRV was calculated using a robust geometric method based on beat-to-beat variation through 10 sec analysis window centred on relative RR interval, which is insensitive to outliers and changes in absolute HR *(1)*.

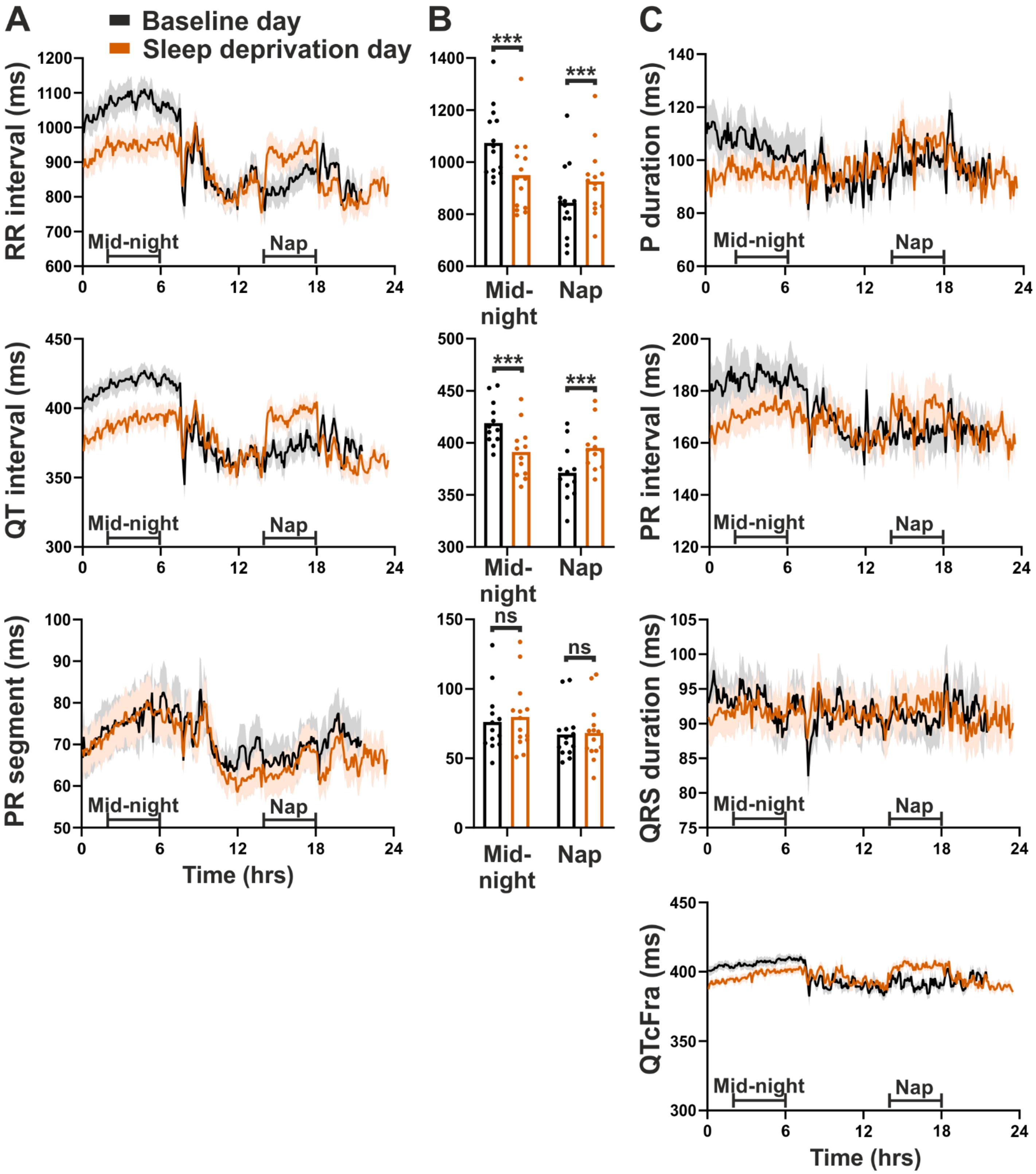

Supplementary Figure 2. ECG parameters recorded in 4-day in-laboratory study in control (non-shift work) individuals (related to Figure 1). A. RR, QT, and PR_{seg} intervals recorded over baseline (black) and total sleep deprivation (TSD, orange) days. shaded region represents +/- SEM. **B.** Bar charts reflect mean RR, QT, and PR_{seg} intervals determined across 'mid-night' and 'nap' windows on baseline and sleep deprivation days (ns $p > 0.05$, ***$p<0.001$, Two-way RM ANOVA/Mixed model). **C**. P wave duration, PR interval, QRS interval, and heart rate adjusted QT (Framingham correction, QTcFra) rhythms recorded over baseline and TSD days. Shaded regions represent +/- SEM. Individual traces were excluded from waveforms where data coverage fell <70% of the 5-min time bins; n (baseline/TSD) = 13/14 (RR), 11/12 (QT), 13/14 (PR_{seg}), 11/13 (P duration), 12/13 (PR_{int}), 12/14 (QRS), 11/12 (QTcFra). For mean night vs nap window analyses n = 14 (RR), 12 (QT), 13 (PR_{seg}). Source data and statistical details are provided as a Source Data File.

	Wehrens et al., 2012							
	Control group Baseline day		Shift group Baseline day		Control group TSD day		Shift group TSD day	
	Mean	SD	Mean	SD	Mean	SD	Mean	SD
**Heart rate (bpm)**	67.8	8.9	67.3	7.1	69.6[a]	9.3	69.8[a]	6.5
**RR interval (ms)**	917.7	128.0	915.8	93.8	887.6[a]	128.9	877.1[a]	79.6
**QT interval (ms)**	385.7	20.9	379.4	22.7	379.8	22.2	370.5[a]	20.1
**QTc interval (ms)**	397.6	11.7	391.7	10.4	396.9	8.5	389.3	11.8
**PR interval (ms)**	156.0	23.1	154.7	16.8	162.3	16.8	153.5	21.9
**PR segment (ms)**	71.9	18.3	64.4	17.9	71.0	18.0	65.6	19.6
**P wave duration (ms)**	82.0	13.9	87.6	6.5	87.1	9.5	82.5	14.3
**QRS duration (ms)**	90.0	8.5	97.2	11.6	89.5	11.1	95.3	11.5

	Wehrens et al., 2012							
	Control				Shift			
	Amplitude (ms)		Peak time (hrs)		Amplitude (ms)		Peak time (hrs)	
	Mean	SD	Mean	SD	Mean	SD	Mean	SD
**RR interval**	106.1	29.7	3.8	0.8	132.8	102.7	3.3	2.1
**QT interval**	25.3	8.4	2.9	1.9	24.7	9.2	3.3	1.6
**PR segment**	7.7	5.6	5.9	4.3	6.1	5.1	6.1	6.2

Supplementary Table 1. ECG parameters in control subjects and experienced shift-workers across baseline and shift days in study 1. Top: ECG parameter means (and standard deviation, SD) across the baseline and TSD days. [a] baseline vs TSD, $p<0.05$; Two-way ANOVA (RM where appropriate). **Bottom:** Rhythmic characteristics derived from baseline recording in control and shift-work groups. Amplitude and time of rhythm peak were calculated by cosinor analysis, with mean and SD taken between subjects (circular mean and SD for peak time). No parameters were significantly different.

	Skene et al., 2018							
	Day condition Day 1 (baseline)		Night condition Day 1 (baseline)		Day condition Day 4		Night condition Day 4	
	Mean	SD	Mean	SD	Mean	SD	Mean	SD
**Heart rate (bpm)**	67.0	9.6	65.4	4.8	68.7	9.4	66.0	8.1
**RR interval (ms)**	938.6	136.2	950.1	72.7	906.3	123.7	942.8	126.3
**QT interval (ms)**	402.2	30.3	402.0	18.3	397.7	31.4	398.7	19.6
**QTc interval (ms)**	411.7	21.8	409.7	17.9	412.1	19.4	407.6	20.4
**PR interval (ms)**	156.1	20.8	157.2	17.7	156.5	18.7	157.6	19.7
**PR segment (ms)**	71.3	18.2	80.1	29.0	71.4	16.1	79.9	32.6
**P wave duration (ms)**	85.3	9.1	83.7	5.5	85.2	8.8	84.6	5.7
**QRS duration (ms)**	67.9	5.4	69.9	9.3	67.9	5.7	71.4	11.1

Supplementary Table 2. ECG parameters measured in 6-day in-laboratory study under day- or night-shift conditions. ECG parameter means (and standard deviation, SD) measured across the baseline day (Day 1) and 4th day of shifted behavioural routine. No significant differences were observed between groups or in response to shifted routine (Two-way ANOVA with RM, $p>0.05$).

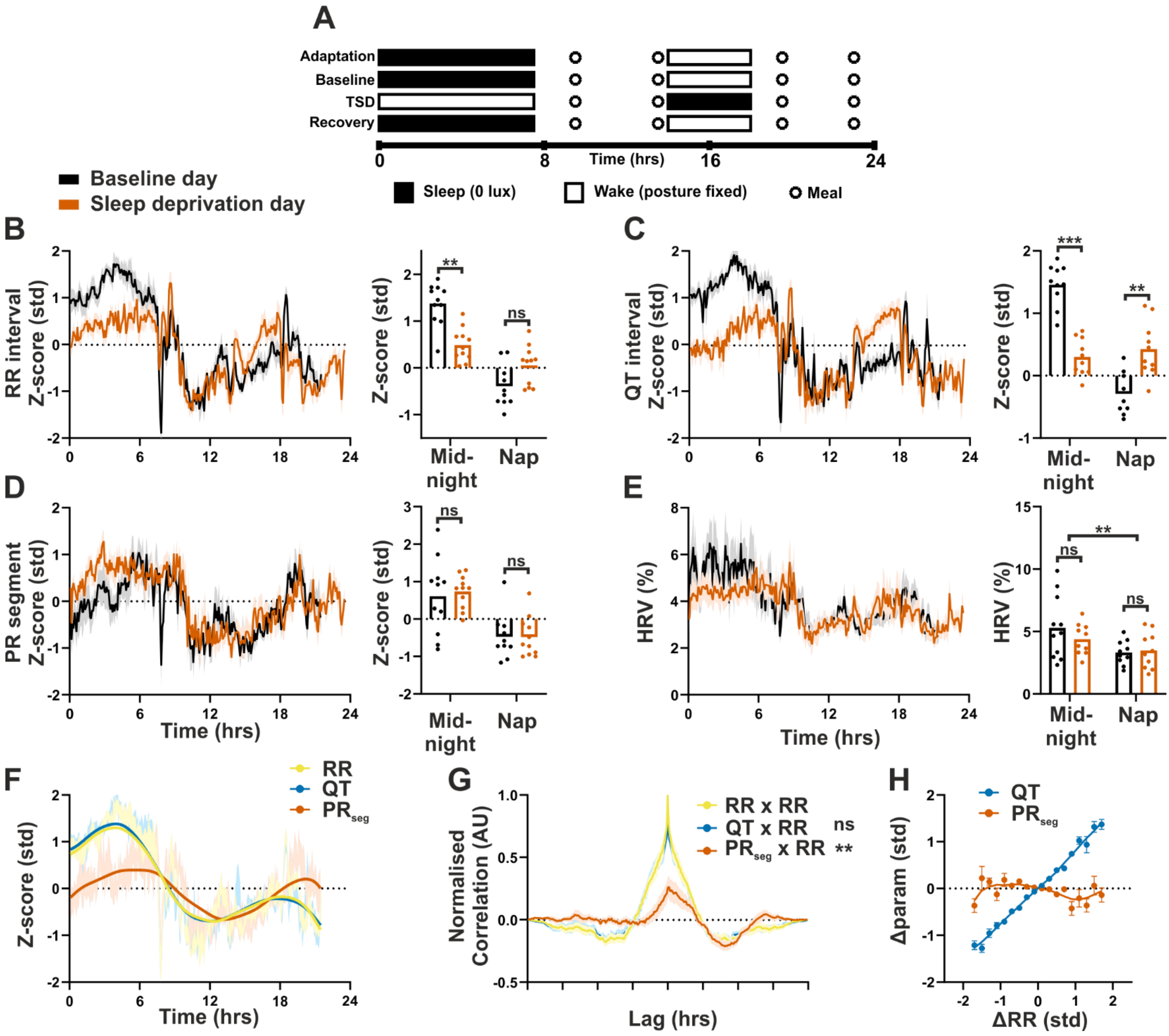

Supplementary Figure 3. ECG parameters recorded in 4-day in-laboratory study in experienced shift-workers (>5 years). A. Schematic of laboratory session (as in Figure 1). **B-E**. Baseline rhythms in RR (**B**), QT (**C**), PR_{seg} (**D**) intervals and HRV (**E**) intervals recorded over baseline (black) and total sleep deprivation (TSD, orange) days. Bar charts reflect mean RR, QT, and PR_{seg} intervals and HRV determined across 'mid-night' and 'nap' windows on baseline and sleep deprivation days (Two-way RM Mixed-Model). Shading represent +/-SEM. Individual traces were excluded from waveform averages where data coverage fell <70% of the 5-min time bins; n (baseline/TSD) = 9/11 (B), 8/10 (C), 7/11 (D), 9/9 (E). For mean night vs nap window analyses, n = 11 (B,D), 10 (C,E). **F.** LOWESS fit to z-scored profiles of RR, QT, and PR_{seg} intervals at baseline. **G.** Cross-correlation between QT and RR intervals, PR segment and RR interval and RR interval autocorrelation (n = 8 QT/RR, 7 PR_{seg}/RR, 9 RR/RR). AU: arbitrary units. **H**. Acute changes in RR were mirrored by concordant changes in QT, but not PR_{seg} (based on 5 min analysis bins; ΔRR reflects z-scored difference in RR between sequential 5-min analysis bins; Δparam reflects concurrent change in QT or PR). Data presented as mean ± SEM. ns p>0.05,*p<0.05, **p<0.01, ***p<0.001. Source data and statistical details are provided as a Source Data File.

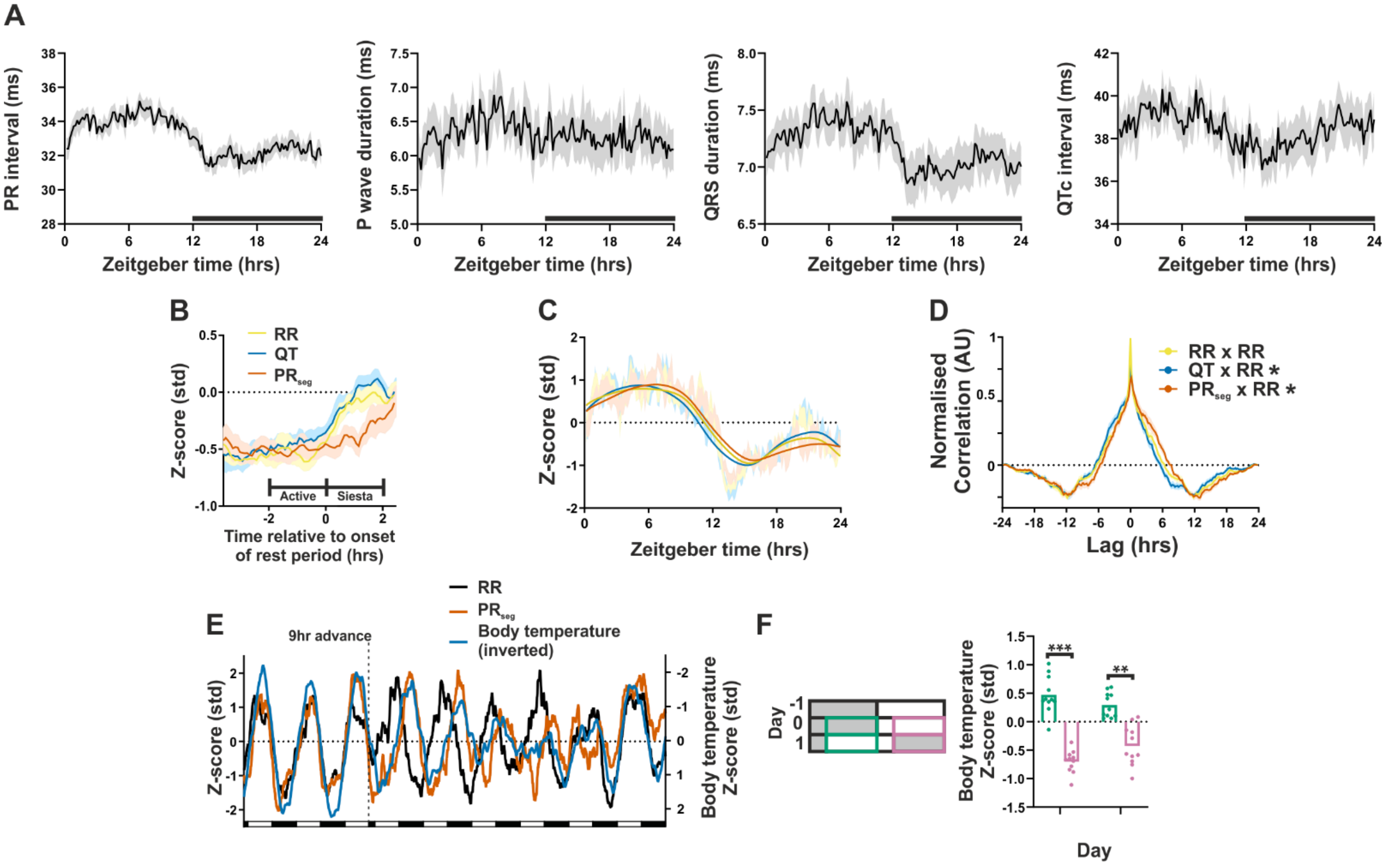

Supplementary Figure 4. Additional ECG parameters and analyses in wild-type mice. A. PR interval, P wave duration, QRS duration and HR-corrected QT interval *(2)* rhythms (related to Figure 3; shading represents +/-SEM; n = 10 mice). **B**. RR (yellow), QT (blue) and PR_{seg} (orange) aligned to the cessation of locomotor activity at the start of the dark phase 'siesta'. Black bars indicate "active" and "rest" periods used for analysis in Fig. 3B. Z-scoring is based on the 5 day recording window for each mouse. **C**. LOWESS fit of RR (yellow), QT (blue) and PR_{seg} (orange) under normal LD conditions. **D**. Phase shift revealed by cross-correlation analyses of RR vs QT and RR vs PR_{seg} (Gaussian fit with one sample t-test; AU: arbitrary units). **E**. RR, PR_{seg} and body temperature from a representative animal across a 9hr phase advance, similar to Fig 3G. Data has been z scored and body temperature inverted for ease of comparison. **F**. Population data for body temperature during the dark (green) and light (pink) phase the day before (day 0) and day of (day 1) the phase advance (Two way RM ANOVA). n = 10 mice (A-D); 11 mice (E,F). *p<0.05, **p<0.01, ***p<0.001. Source data and statistical details are provided as a Source Data File.

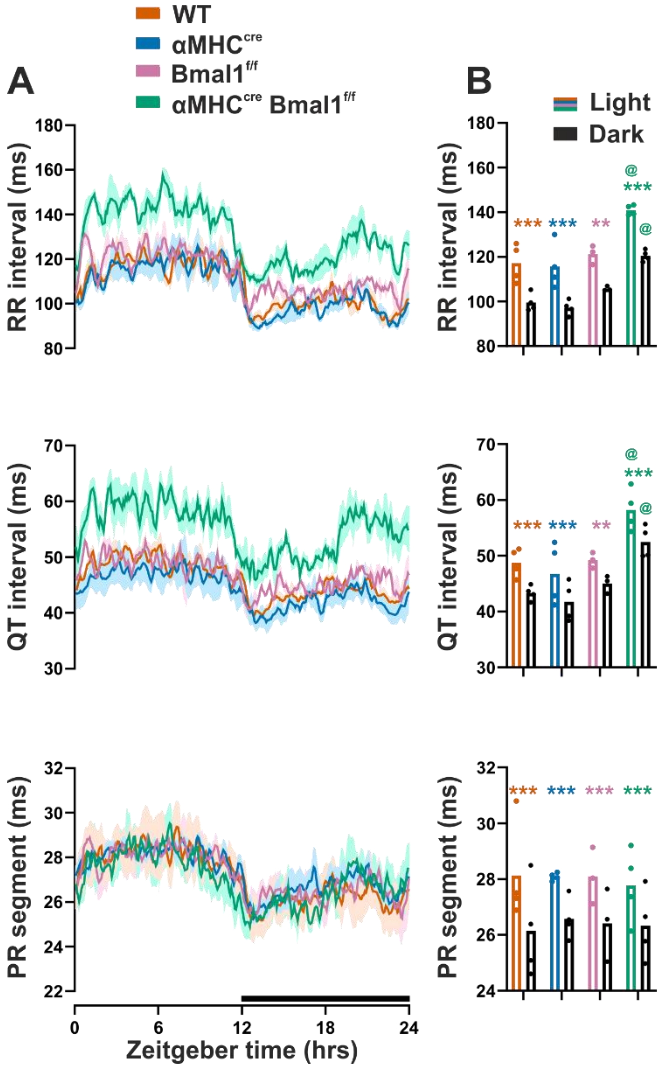

Supplementary Figure 5. **ECG profiles in cardiomyocyte-specific *Bmal1* deleted mice and appropriate control mouse lines. A.** Daily ECG parameter rhythms recorded from wild-type (C57B6J), *αMHC^{cre}*, *Bmal1$^{fl/fl}$* and *αMHC^{cre}Bmal1$^{fl/fl}$*. Black bars represent darkness. Shaded regions represent SEM. **B**. Quantification of ECG parameters (genotype mean) across the light and dark phases of the day. Coloured asterisks indicate significant time of day difference; @ symbols indicate significant difference between *αMHC^{cre}Bmal1$^{fl/fl}$* and all other genotypes at equivalent light/dark phase (Two-way RM ANOVA, Sidak's post hoc). ns p>0.05, *p<0.05, **p<0.01, ***p<0.001. n = 4 mice (WT, *Bmal$^{Fl/Fl}$*, *aMHC^{cre}Bmal$^{Fl/Fl}$*), n = 3 mice (*aMHC^{cre}*). Source data and statistical details are provided as a Source Data File.

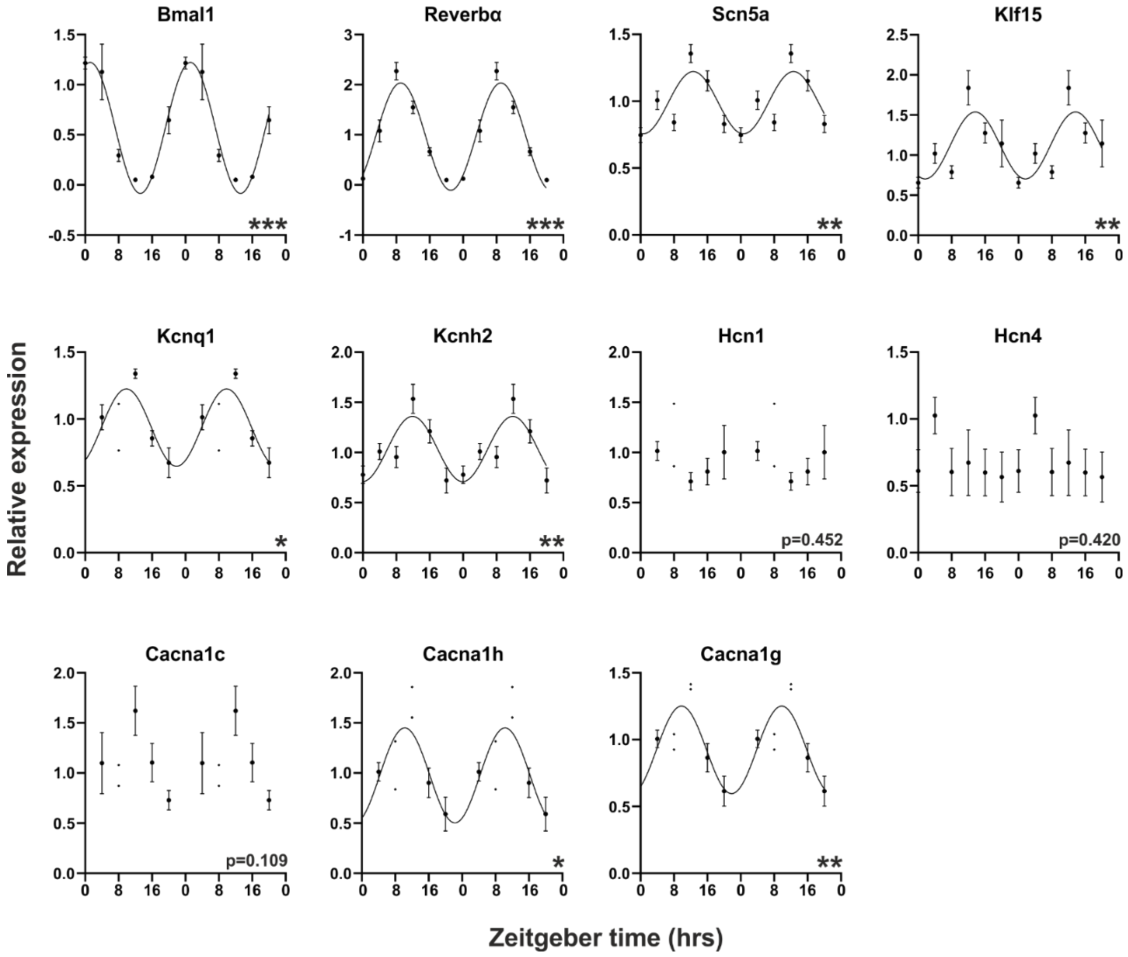

Supplementary Figure 6. Diurnal rhythms in SA node gene expression. qPCR of clock genes (Bmal1 and Reverbα) and ion channels across 24 hr reveals widespread rhythms in gene expression. The sodium channel Scn5a, potassium channels (Kcnq1, Kcnh2) and related transcription factor (Klf15) and T-type calcium channels (Cacna1h, Cacna1g) all display robust rhythmicity, determined by cosinor analysis (24 hr constrained sine wave vs horizontal line; *$p<0.05$, **$p<0.01$, ***$p<0.001$; n=2-4 biologically independent samples/time-point). Data have been double plotted for visualisation only. Source data and statistical details are provided as a Source Data File.

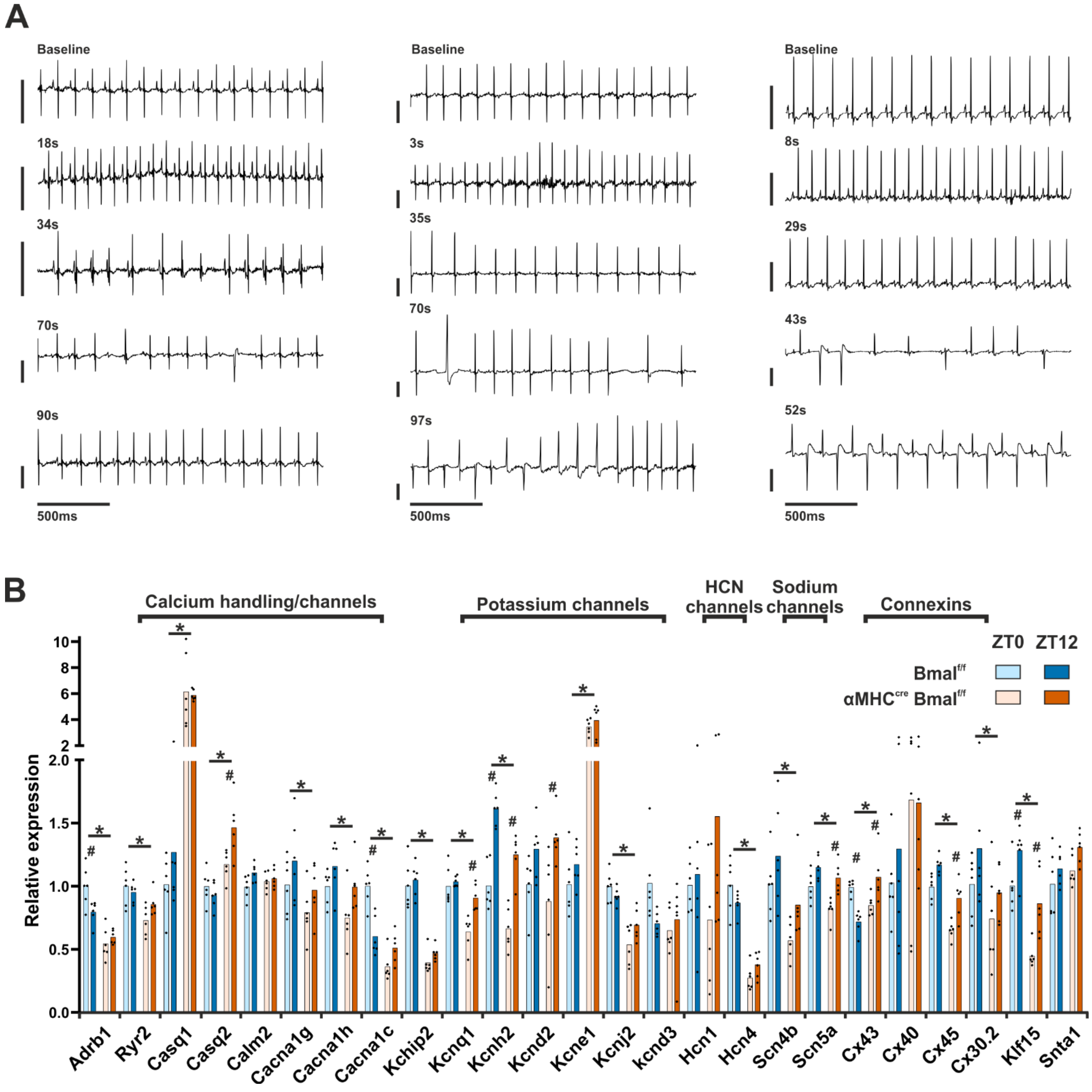

Supplementary Figure 7. qPCR of arrhythmia-related genes reveals potential mechanisms of arrhythmogenesis. **A**. Example traces from *αMHC^{cre} Bmal1$^{f/f}$* animals after in-vivo arrhythmia induction, similar to Fig 6. Left: no evidence of BVT. Middle: short bout of BVT (~6 s), Right: strong evidence of BVT. Vertical scale bars represent 0.5 mV and inset times are time from returning to cage after injection. **B**. qPCR of arrhythmia-associated genes from *Bmal1$^{f/f}$* (blue) and *αMHC^{cre} Bmal1$^{f/f}$* (orange) ventricles collected at ZT0 (light colour) and ZT12 (dark colour). Data presented relative to *Bmal1$^{fl/fl}$* ZT0 group (Two way ANOVA, Sidak's MC adjusted for multiple testing; n=6 biologically independent samples/group). Asterisks indicate genotype difference ($p<0.05$) while hashes represent post hoc time of day difference ($p<0.05$). Source data and statistical details are provided as a Source Data File.

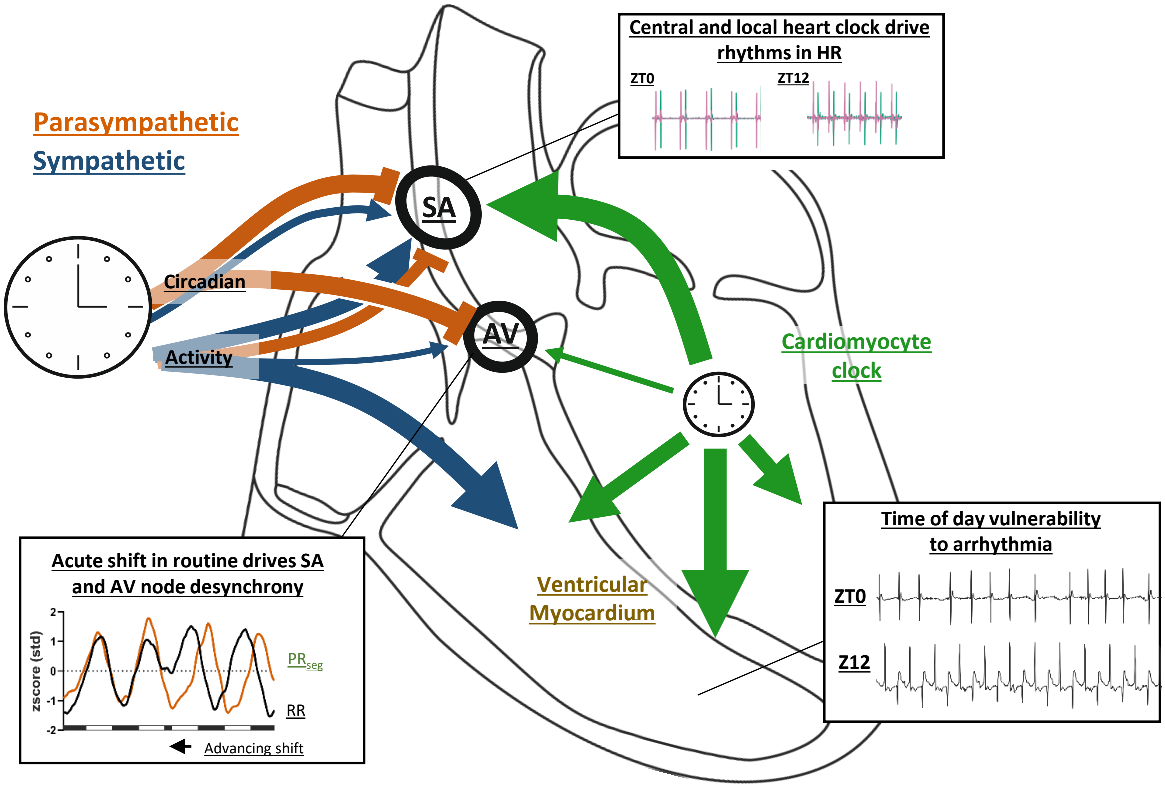

Supplementary Figure 8. Schematic of circadian and non-circadian influences over cardiac electrophysiology. Local to the heart, the cardiomyocyte clock drives rhythms in firing rate and cellular excitability within the SA node and ventricular myocardium. Multiple distinct pathways from the brain, segregating acute arousal state changes from longer term circadian inputs deliver rhythmic information to the heart. While the SA node is influenced by both arousal state and a circadian input of neural origin, the AV node is disproportionately sensitive to the circadian input and relatively insensitive to arousal state. There is clear evidence for differential innervation and functional control over sinus rhythm and AV nodal conduction at the level of parasympathetic ganglia *(3–7)* and preganglionic nuclei of the brainstem *(8,9)*. Arousal state manipulations, as well as changing sinus rate, dramatically alter ventricular repolarisation time, either through direct autonomic innervation or indirectly via changes in HR. While ventricular repolarisation time is very rhythmic, we see a markedly reduced diurnal amplitude when corrected for HR, suggesting that repolarisation of the ventricular myocardium is predominantly driven passively by the rhythm in HR, while direct circadian input from the brain is less influential.

Supplementary Table 3. qPCR Primer sequences.

	Sequence	
**Gene**	Forward	Reverse
**Ppib**	GGAGATGGCACAGGAGGAAA	CCGTAGTGCTTCAGTTTGAAGTTCT
**Actb**	CTGTCCCTGTATGCCTCTG	ATGTCACGCACGATTTCC
**Hprt**	GTTGGATACAGGCCAGACTTTGTTG	GATTCAACTTGCGCTCATCTTAGGC
**Bmal1**	GTCGAATGATTGCCGAGGAA	GGGAGGCGTACTTGTGATGTTC
**Reverbα**	GTCTCTCCGTTGGCATGTCT	CCAAGTTCATGGCGCTCT
**Scn4b**	ACCATCTACGCTATTAACGGCT	CGCTGTTATTGTAGGACCACTT
**Scn5a**	AGACTTCCCTCCATCTCCAGATA	TGTCACCTCCAGAGCTAGGAAG
**Klf15**	CAAGAGCAGCCACCTCAAG	GACACTGGTACGGCTTCACA
**Kchip2**	AGCGTGGAGGATGAGTTTGAAC	TTCCCCGAAGAATCACTGACA
**Kcnq1**	CATCGGTGCCCGTCTGAACAGG	TTGCTGGGTAGGAAGAGCTCAG
**Kcnh2**	GATCGCCTTCTACCGGAAA	CATTCTTCACGGGTACCACA
**Kcnd2**	TCGCCCATCAAGTCACAGTC	CTGGAGGTGTTGGGATGCTT
**Kcnd3**	AGCTTCCGTCAGACCATGTG	GGCAAAAGAAAGCCACCGAAT
**Kcne1**	CTGCCCAATTCCACGACTGTT	GAGCTGAGACTTACGAGCCA
**Kcnj2**	CACAGCTTCTCAAATCTAGGATCA	CTATTTCGTGAACGATAGTGATGG
**Hcn1**	CTCTTTTTGCTAACGCCGAT	CATTGAAATTGTCCACCGAA
**Hcn4**	GCATGATGCTTCTGCTGTGTCACT	TTCACCATGCCATTGATGGACACC
**Cacna1c**	TCCCGAGCACATCCCTACTC	ACTGACGGTAGAGATGGTTGC
**Cacna1h**	GCAGCCATCCTCGTCAATAC	AGCATCTCCAAGGCAAACAT
**Cacna1g**	TCCTTGGAGATGCTGCTGAAG	CACACGCTGATGACCACAATG
**Cacna1d**	GCTTACGTTAGGAATGGATGGAA	GAAGTGGTCTTAACACTCGGAAG
**Adrb1**	CTCATCGTGGTGGGTAACGTG	ACACACAGCACATCTACCGAA
**Ryr2**	TGCTTCATCTGTGGGATTGG	CCTGTGTGTTCTGTTTCATCC
**Casq1**	CCTCCCAAGTCTCGTACATAC	AGCCTATGACCATCCCAGA
**Casq2**	AAGCCTGTATGTTCTGAAGGGT	CTGCCTCTTGGAATGCTTTGTA
**Calm2**	ACGGGGATGGGACAATAACAA	TGCTGCACTAATATAGCCATTGC
**Cx40**	TTTGGCAAGTCACGGCAGGG	TGTCACTATGGTAGCCCTGAG
**Cx43**	CCCCACTCTCACCTATGTCTCC	ACTTTTGCCGCCTAGCTATCCC
**Cx45**	GGGAAAGCAACAAACAAAGT	AAAGGCATCATAGCAGACATT
**Cx30.2**	TGATCATGCTGATCTTCCGCATCC	GCTGCAACGTGTTACACACGAACT
**Snta1**	CCAGACGGAGGCCCTTTTT	GCCTGTACCGCTTCATCGT

Supplementary references

1. Vollmer, M. A robust, simple and reliable measure of heart rate variability using relative RR intervals. *Comput. Cardiol. (2010).* **42,** 609–612 (2015).
2. Roussel, J., Champeroux, P., Roy, J., Richard, S., Fauconnier, J., Le Guennec, J. Y., Thireau, J. The Complex QT/RR Relationship in Mice. *Sci. Rep.* **6,** 1–9 (2016).
3. Gatti, P. J., Johnson, T. A., Phan, P., Jordan, I. K., Coleman, W., Massari, V. J. The physiological and anatomical demonstration of functionally selective parasympathetic ganglia located in discrete fat pads on the feline myocardium. *J. Auton. Nerv. Syst.* **51,** 255–259 (1995).
4. Randall, W. C., Ardell, J. L., Calderwood, D., Milosavljevic, M., Goyal, S. C. Parasympathetic ganglia innervating the canine atrioventricular nodal region. *J. Auton. Nerv. Syst.* **16,** 311–323 (1986).
5. Randall, W. C., Ardell, J. L., Wurster, R. D., Milosavljevic, M. Vagal postganglionic innervation of the canine sinoatrial node. *J. Auton. Nerv. Syst.* **20,** 13–23 (1987).
6. Billman, G. E., Hoskins, R. S., Randall, D. C., Randall, W. C., Hamlin, R. L., Lin, Y. C. Selective vagal postganglionic innervation of the sinoatrial and atrioventricular nodes in the non-human primate. *J. Auton. Nerv. Syst.* **26,** 27–36 (1989).
7. Blinder, K. J., Gatti, P. J., Johnson, T. A., Lauenstein, J. M., Coleman, W. P., Gray, A. L., Massari, V. J. Ultrastructural circuitry of cardiorespiratory reflexes: There is a monosynaptic path between the nucleus of the solitary tract and vagal preganglionic motoneurons controlling atrioventricular conduction in the cat. *Brain Res.* **785,** 143–157 (1998).
8. Gatti, P. J., Johnson, T. A., Massari, V. J. Can neurons in the nucleus ambiguus selectively regulate cardiac rate and atrio-ventricular conduction? *J. Auton. Nerv. Syst.* **57,** 123–127 (1996).
9. Geerling, J. C., Shin, J. W., Chimenti, P. C., Loewy, A. D. Paraventricular hypothalamic nucleus: Axonal projections to the brainstem. *J. Comp. Neurol.* **518,** 1460–1499 (2010).
