# Supplementary material for: Distinct circadian mechanisms govern cardiac rhythms and susceptibility to arrhythmia
Source: Nat Commun. 2021 Apr 30;12:2472. doi: 10.1038/s41467-021-22788-8 (PMC8087694; doi:10.1038/s41467-021-22788-8)
Supplement: Supplementary file 3 — Reporting Summary [file 41467_2021_22788_MOESM3_ESM.pdf]

nature research

Corresponding author(s): David A Bechtold

Last updated by author(s): Feb 1, 2021

Reporting Summary

Nature Research wishes to improve the reproducibility of the work that we publish. This form provides structure for consistency and transparency in reporting. For further information on Nature Research policies, see our Editorial Policies and the Editorial Policy Checklist.

Statistics

For all statistical analyses, confirm that the following items are present in the figure legend, table legend, main text, or Methods section.

n/a	Confirmed	
☐	☒	The exact sample size (*n*) for each experimental group/condition, given as a discrete number and unit of measurement
☐	☒	A statement on whether measurements were taken from distinct samples or whether the same sample was measured repeatedly
☐	☒	The statistical test(s) used AND whether they are one- or two-sided<br>*Only common tests should be described solely by name; describe more complex techniques in the Methods section.*
☒	☐	A description of all covariates tested
☐	☒	A description of any assumptions or corrections, such as tests of normality and adjustment for multiple comparisons
☐	☒	A full description of the statistical parameters including central tendency (e.g. means) or other basic estimates (e.g. regression coefficient) AND variation (e.g. standard deviation) or associated estimates of uncertainty (e.g. confidence intervals)
☐	☒	For null hypothesis testing, the test statistic (e.g. *F*, *t*, *r*) with confidence intervals, effect sizes, degrees of freedom and *P* value noted<br>*Give P values as exact values whenever suitable.*
☒	☐	For Bayesian analysis, information on the choice of priors and Markov chain Monte Carlo settings
☒	☐	For hierarchical and complex designs, identification of the appropriate level for tests and full reporting of outcomes
☒	☐	Estimates of effect sizes (e.g. Cohen's *d*, Pearson's *r*), indicating how they were calculated

Our web collection on statistics for biologists contains articles on many of the points above.

Software and code

Policy information about availability of computer code

Data collection	Data Sciences International (Ponemah system)<br>ProFusion PSG 2 (version 2.1 Compumedics Ltd) (study 1)<br>CardioScan software (version 11.4, Stateline) (study 2)
Data analysis	Commercial software used:<br>GraphPad Prism v8.4.0<br>MATLAB R2018a<br>DSI radio telemetry Ponemah system<br>Powerlab (4/35) and LabChart (v8) ADinstruments<br>Multichannel experimenter version 2.8.2.18079<br>Multichannel analyzer 2.13.5.19246<br><br>Custom software used:<br>Code for ECG analyses is publicly available through GitHub<br>https://github.com/EdHayter/Hayter-et-al.-ECG-analysis<br>https://doi.org/10.5281/zenodo.4483853

For manuscripts utilizing custom algorithms or software that are central to the research but not yet described in published literature, software must be made available to editors and reviewers. We strongly encourage code deposition in a community repository (e.g. GitHub). See the Nature Research guidelines for submitting code & software for further information.

Data

Policy information about availability of data

All manuscripts must include a data availability statement. This statement should provide the following information, where applicable:

- Accession codes, unique identifiers, or web links for publicly available datasets
- A list of figures that have associated raw data
- A description of any restrictions on data availability

The datasets generated during the current study are available from the corresponding author on reasonable request. Source data for the figures and statistical analyses details are included with this article.

Field-specific reporting

Please select the one below that is the best fit for your research. If you are not sure, read the appropriate sections before making your selection.

☒ Life sciences ☐ Behavioural & social sciences ☐ Ecological, evolutionary & environmental sciences

For a reference copy of the document with all sections, see nature.com/documents/nr-reporting-summary-flat.pdf

Life sciences study design

All studies must disclose on these points even when the disclosure is negative.

Sample size	Sample sizes for human and animal studies were based on extensive previous experience (e.g. West et al., 2017 (doi: 10.1038/s41467-017-00462-2); Cunningham et al., 2016 (doi: 10.1038/srep29983); Hand et al., 2015 (doi: 10.2337/db13-1835)) and appropriate power calculation.
Data exclusions	In Human Study 1, four subjects were excluded from all QT analyses and comparisons due to inability to accurately measure QT interval. Two subjects were in the control group and 2 were in the shift-work group. This has been stated in the methods. For daily profiles of ECG parameters (in Figures 1, S2, S3), individual traces were excluded where data coverage fee below 70% of the time-bins. Resulting n numbers have been made clear in the appropriate figure legends.
Replication	Human studies involved two independent studies undertaken at separate research institutions. However, these did not constitute dirct replication, as the study designs were different.<br><br>For animal experiments, careful consideration was given to experimental design to optimization of animal use. Animal experiments were therefore performed only once. However, some studies were independently repeated due to separate studies on wildtype C57B6J mice and transgenic lines, with consistent results across these studies. This included circadian profiling of ECG measures, in vivo autonomic blockade, and ex vivo isolated heart preparations.
Randomization	Human Studies:<br>In human study 1, no randomisation was required as all participants underwent an the same procedure. In human study 2, The participants were assigned at random to either the simulated day shift condition (7 participants) or the simulated night shift condition (7 participants).<br><br>Animal Studies:<br>Inherent randomization in housing and group allocation was provided by breeding (all our studies using transgenic animals employ heterozygotic breeding and littermate control/transgenic mice for experimental study). Additional group allocation was determined age matching of mice prior to experimentation.
Blinding	Blinding was provided by number coding of ECG traces and samples prior to analyses. ECG analyses were automated. Sample coding continued through analyses and histological assessment, with genotype and group revealed only after completion.<br>In human studies, subjects ids were coded and not assigned to group until after ECG extraction and analyses were performed.

Reporting for specific materials, systems and methods

We require information from authors about some types of materials, experimental systems and methods used in many studies. Here, indicate whether each material, system or method listed is relevant to your study. If you are not sure if a list item applies to your research, read the appropriate section before selecting a response.

Materials & experimental systems

n/a	Involved in the study
☒	☐ Antibodies
☒	☐ Eukaryotic cell lines
☒	☐ Palaeontology and archaeology
☐	☒ Animals and other organisms
☐	☒ Human research participants
☒	☐ Clinical data
☒	☐ Dual use research of concern

Methods

n/a	Involved in the study
☒	☐ ChIP-seq
☒	☐ Flow cytometry
☒	☐ MRI-based neuroimaging

Animals and other organisms

Policy information about studies involving animals; ARRIVE guidelines recommended for reporting animal research

Laboratory animals	Mus musculus C57BL/6J males were used (transgenic models described in text). Mice were aged 10-16 weeks at time of experiment. Animal unit conditions: temperature 22C +/- 2C; Humidity 52% +/- 7%; food and water were provided ad libitum; ambient light:dark cycle is defined by experiment in the methods section and/or figure legends.
Wild animals	The study did not involve wild animals.
Field-collected samples	The study did not involve samples collected from the field.
Ethics oversight	The University of Manchester Animal Welfare and Ethical Review Body (AWERB) approved all procedures. All animal experiments were licensed under the UK Animals (Scientific Procedures) Act 1986.

Note that full information on the approval of the study protocol must also be provided in the manuscript.

Human research participants

Policy information about studies involving human research participants

Population characteristics	Study 1 consisted of 14 control and 11 long-term (>5yr) shift workers, all male aged between 25-45 years. All participants were healthy and had written consent from their general practitioner to participate.<br>Study 2 consisted of 10 male and 4 female volunteers aged 22-35 years. Participants were physically and mentally healthy and assigned into 'day' and 'night' shift groups at random. Full details for these trials have been previously reported (Wehrens et al 2010 ,2012; Skene et al 2018; Skornyakov et al 2019).
Recruitment	Study 2. Individuals aged 22-40 were recruited from the general population in the greater Spokane (Wash.) area, through print and online advertisements and flyers. Interested individuals contacted the laboratory and underwent a brief telephone interview and subsequent in-laboratory screening session to obtain informed consent and verify eligibility based on the predetermined inclusion criteria for the study. Study 1. Individuals were recruited from the local area. If they passed predetermined eligibility criteria at screening, they were invited to participate. We do not believe that potential self-selection bias are likely to have substantially impacted our results.<br>For more information please see references above.
Ethics oversight	University of Surrey (Surrey, UK) and University of Washington (WA, USA)

Note that full information on the approval of the study protocol must also be provided in the manuscript.
